# Uptake of Gaseous Elemental Mercury by a Rainforest: Insights from a Tropical Glasshouse Used as a Dynamic Flux Chamber

**DOI:** 10.1021/acs.est.5c05823

**Published:** 2025-08-26

**Authors:** Basil Denzler, Werner Eugster, Christian Bogdal, Kevin Bishop, Nina Buchmann, Konrad Hungerbühler, Stefan Osterwalder

**Affiliations:** a Institute for Chemical and Bioengineering, Department of Chemistry and Applied Biosciences, ETH Zurich, Zurich 8093, Switzerland; b 30942 Kantonsschule im Lee, Winterthur 8400, Switzerland; c Department of Environmental System Sciences, Institute of Agricultural Sciences, ETH Zurich, Zurich 8092, Switzerland; d Zurich Forensic Science Institute, Zurich 8004, Switzerland; e Department of Aquatic Sciences and Assessment, Swedish University of Agricultural Sciences, Uppsala 75007, Sweden; f Hydrology and Climate, Department of Geography, 124130 University of Zurich, Zurich 8057, Switzerland

**Keywords:** tropics, forest, flux, deposition, GPP, CO_2_

## Abstract

Vegetation uptake of gaseous elemental mercury (Hg^0^) is the main deposition pathway to terrestrial environments. However, the fluxes and processes of forest-atmosphere Hg^0^ exchange remain ill-constrained, especially in rainforests. To help address this, we used the 1 ha Masoala Rainforest hall of the Zoo Zurich as a dynamic flux chamber to measure net rainforest Hg^0^ fluxes and even calibrate Hg^0^ deposition velocities with turbulence measurements. The net Hg^0^ flux correlated well with CO_2_ assimilation, showing peak Hg^0^ uptake at noon. The interquartile range of Hg^0^ uptake spanned from 1.69 to 3.45 ng m^–2^ h^–1^ during the day and from 0.01 to 0.44 ng m^–2^ h^–1^ at night. The study results revealed a Hg^0^-specific canopy resistance (*R*_c_ = 1000 s m^–1^), which underlined the importance of stomatal uptake as a dominant Hg^0^ deposition pathway in the rainforest. Even though a dynamic flux chamber, however large, is only an approximation of a real rainforest, our findings underline the value of whole-rainforest flux studies both for constraining Hg exchange with the atmosphere and resolving the role of specific mechanisms.

## Graphical abstract

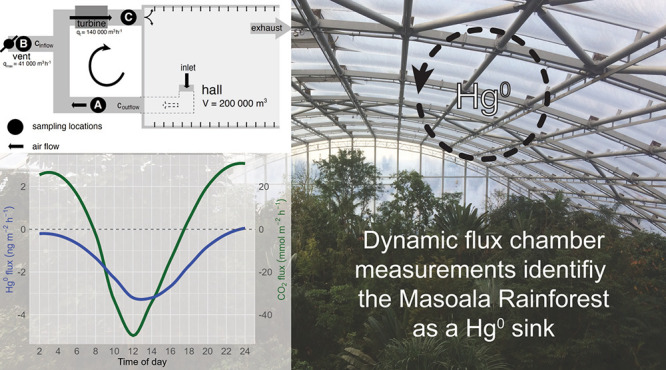

## 1 Introduction

Mercury (Hg) is a toxic metal ubiquitously present in all environmental systems.^1^ The largest reservoir for Hg is the Earth’s crust, where it mainly occurs as a stable mineral.^2^ In the absence of human activities, mercury (Hg) was mobilized by geological sources, primarily volcanic emissions.^3^ Today, anthropogenic emissions, mainly from artisanal or small-scale gold mining and coal combustion, have increased the atmospheric Hg pool by about 450% compared to the 15th century^4^ Gaseous elemental Hg (Hg^0^) typically accounts for more than 90% of that total atmospheric Hg pool^5^ and has an atmospheric lifetime between 2 and 13 months.^6–8^ In the atmosphere, Hg^0^ is subject to long-range transport and deposition far from emission sources.^9^ Dry deposition is the most important atmospheric Hg deposition pathway to terrestrial ecosystems:^10^ Hg^0^ is taken up by foliage and reaches the soil via litterfall.^11^ Thus, litterfall has been used as a proxy for atmospheric Hg^0^ deposition in forests.^12^ Globally, litterfall provides about 50% (1180–1410 Mg per year) of the total Hg inputs to land and is more important than dry deposition of Hg^0^ to bare soil.^13^ Dry deposition of reactive Hg (Hg^II^) to foliage surfaces and soil, as well as wet deposition of Hg^II^ with rain and snow (incl. wash-off from leaves and needles via throughfall), constitute the remaining fraction of the total Hg deposition to terrestrial ecosystems.^14,15^

Forest ecosystems are recognized as the largest terrestrial sink for atmospheric Hg^0^.^16–18^ Stable Hg isotope analysis has revealed that about 90% of the total Hg in foliage originates from atmospheric Hg^0^.^19–23^ Foliage assimilates Hg^0^ in two ways, via stomatal gas exchange^11,24–26^ and nonstomatal Hg^0^ uptake.^27,28^ Stomatal Hg^0^ uptake in broadleaf foliage is reported to be three times higher than in coniferous needles, and increases under conditions of high physiological activity promoted by factors such as sufficient solar radiation, low water pressure deficits, adequate soil water content and favorable nutrient status.^25^ Nonstomatal Hg^0^ uptake has also been identified from laboratory studies^27,28^ and in situ measurements using dynamic flux bags.^14,22,29,30^ These studies reveal that Hg^0^ uptake can occur not only during the day but also at night, indicating the potential for substantial nonstomatal Hg^0^ uptake by foliage. As an illustrative example, a study performed in a midlatitude coniferous forest, suggested that needle Hg^0^ uptake occurs during the night and accounts for about a quarter of the total Hg^0^ deposition.^31^

The forest-atmosphere Hg^0^ flux constitutes not only Hg^0^ uptake but also photochemically induced Hg^0^ re-emission, i.e., stomatal Hg^0^ re-emission and Hg^0^ re-emission from leaf surfaces,^22^ as well as soil Hg^0^ re-emission driven by biotic and abiotic Hg^0^ reduction processes.^32^ To include all of these exchange pathways, net Hg^0^ flux measurements are required which are typically conducted above the forest canopy using micrometeorological techniques.^31,33–37^ However, whole-ecosystem, micrometeorological Hg^0^ flux measurements are methodologically challenging, associated with high costs, and the need for specialized expertise.^38^ Therefore, dynamic flux chambers have been the most frequently applied method for Hg^0^ flux measurements.^39^ These, however, are typically relatively small (ca. 0.03 m^3^) and cannot be applied to ecosystems with high tree canopies.^40–44^ Net forest-atmosphere Hg^0^ flux measurements are therefore scarce. Furthermore, tropical forests are less well represented in the available data, whether it is for the net ecosystem exchange or small chamber studies.

Rainforests ecosystems have a particularly large capacity of Hg^0^ uptake and therefore play a pivotal role in the global Hg cycle:^16^ Tropical and subtropical forests are estimated to contribute 70% to global Hg^0^ litterfall deposition,^45^ and the Amazon rainforest alone takes up 30% of the Hg^0^ that is deposited to terrestrial surfaces.^46^ These estimates, however, are associated with large uncertainties mainly because physiological controls on foliage Hg^0^ uptake are poorly constrained and observational flux data are lacking.^18^

The factors briefly summarized above contribute to the large uncertainties in global estimates of forest-atmosphere Hg^0^ exchange (−727 to 703 Mg a^–1^; 37.5th to 62.5th percentiles).^39^ Those uncertainties are reflected in the range of results from different studies. Net annual Hg^0^ re-emission was detected from a lightly to moderately polluted, subtropical evergreen coniferous forest using the aerodynamic gradient method.^35^ In contrast, subtropical broadleaf forests,^37^ a midlatitude broadleaf forest,^36^ and a midlatitude coniferous forest^31^ were identified as sinks for atmospheric Hg using relaxed eddy accumulation in China and flux-gradient methods in the U.S. It is interesting to note that net Hg^0^ deposition in the midlatitude broadleaf forest was three times greater than Hg^0^ deposition through litterfall.^36^ Such comprehensive mass balance studies draw attention to uncertainties in forest Hg cycling and highlight the need for an improved model parametrization to better assess forest-atmosphere Hg^0^ exchange globally.^47^

While recent estimates of global forest Hg^0^ uptake were adjusted upward to reflect observational and modeling advances,^13^ the accuracy of these estimates is constrained by a number of factors. These include the limited availability of whole ecosystem measurements of net Hg fluxes, ambiguity about the role of stomatal vs nonstomatal uptake in those net fluxes, and the limited representation of rainforests in the available observations.

Controlled, large-scale ecosystem studies are one way of filling gaps in the knowledge of ecosystem behavior, despite the challenges of isolating any ecosystem, much less a tropical rainforest.^48–51^ The Masoala Rainforest hall of the Zoo Zurich provides a unique opportunity to measure net Hg^0^ exchange with the atmosphere over a rainforest by treating the 1 ha tropical glasshouse as a scaled-up flux chamber with a total volume of 200’000 m^3^. The ability to measure turbulence in the hall also creates an opportunity to determine a Hg^0^-specific canopy resistance. The objectives of this study that simultaneously measured net Hg^0^ and CO_2_ fluxes inside the Masoala Rainforest hall were to (i) resolve diel Hg^0^ and CO_2_ flux patterns (ii) determine a cumulative Hg^0^ flux, and (iii) estimate a Hg^0^-specific canopy resistance.

## 2 Materials and Methods

### 2.1 Study Site

The Masoala Rainforest of the Zoo Zurich is one component of the zoo’s conservation strategy. On an area of 10’856 m^2^ within a climate-controlled glasshouse with a height of 30 m, a dense stand of Madagascan rainforest is grown. It is open to visitors between November and February from 10 a.m. to 5 p.m. and between March and October from 10 a.m. to 6 p.m. There are more than 500 plant species growing, from which about 80% can be found in Madagascar. The leaf area index (LAI) of the plants within the hall was approximately 6 m^2^ m^–2^, derived from the plant area index (PAI) of 7.4 ± 4.6 m^2^ m^–2^ defined during tests of equipment for terrestrial laser scanning^52^ and a typical leaf fraction of 0.8, accounting for nonleaf components such as stems and branches. This LAI falls within the range typically observed in tropical rainforests, which commonly exhibit LAI values between 4 and 8 m^2^ m^–2^,^53^ suggesting that the canopy density in the Masoala hall is broadly comparable to that of natural rainforest ecosystems. The Masoala hall is also home to a variety of insects and vertebrates, including fish, amphibians, reptiles, birds and small mammals. The soil in the hall, which was composed according to Malagasy soil properties, is around 50 to 70 cm thick and was deposited on natural soil of the Zürichberg. The lower soil layer mainly consists of lava stone, while the upper layer is made of 20% pumice, 20% zeolite, 30% lava, and 30% clay. The top soil layer is 5 cm thick and composed of long-fiber peat, leaves and branches.^54^ The rainforest stand is periodically watered by a sprinkler system in the roof of the Masoala hall. The roof is constructed from transparent ethylenetetrafluoroethylene (ETFE) copolymer panels through which about 70% of visible light passes. A ventilation system provides circulation flow and fresh air to the Masoala hall (Figure 1). Humidity and temperature inside the hall are regulated to maintain a tropical climate all year round. Two heat exchange elements keep the temperature from dropping below 18 °C. During our measurement period, the average temperature was 22 °C, and heating was activated only during the early morning hours. The air circulation is generated by two powerful turbines. These produce underpressure on the vent side (point A, Figure 1) which draws air from the center of the Masoala hall through a ventilation shaft. Inside the shaft, a lid to the outside is used to regulate the influx of fresh air (point B, Figure 1). The air is then blown back into the Masoala hall through venturi nozzles aligned on both sides along the hall (point C, Figure 1). Any overpressure is released through a hatch. The system can be described as a flow-through, vented, dynamic flux chamber.^55^ Further methodological considerations are discussed in more detail in the Supporting Information S1.

**Figure 1:**
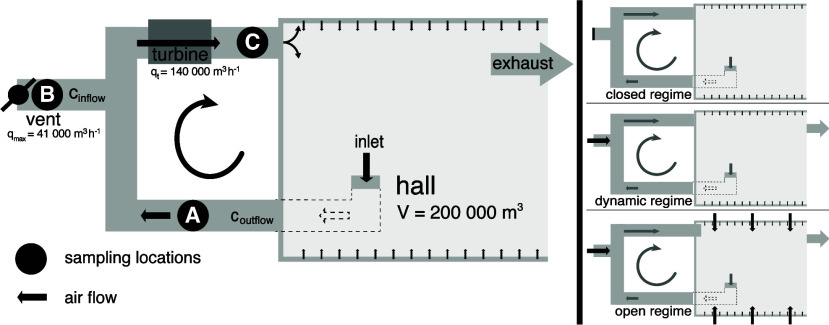
Horizontal scheme of the Masoala hall ventilation system. A vent is used to control the inflow of fresh air into the hall (B). Air circulation within the hall is achieved by pulling air through an inlet at ground level in the center of the hall, mixing it with outside air, and blowing it back into the hall along the walls at about 3 m height through venturi nozzles. The points A, B, and C mark the sampling locations. The location of in- and outflow gas concentration measurements are labeled *c*_inflow_ and *c*_outflow_. The three different operation regimes are shown to the right. The closed regime is characterized by the vent being closed, with the circulation running; the dynamic regime is characterized by the vent being open; the open regime is characterized by additional flaps at the top and alongside the hall being open.

### 2.2 Operation Regimes of the Masoala Hall

The Masoala hall has three different operation regimes: closed, dynamic, and open (Figure 1). The “closed regime” corresponds to a nonvented, dynamic flux chamber. It operates during cold winter periods, especially during the nights. The vent is closed completely, and no fresh air enters the Masoala hall. Air is constantly drawn through the inlet and pumped back into the hall along the sidewalls to generate turbulence. During warmer periods or when humidity gets too high, the “dynamic regime” is used where the degree of vent opening within the ventilation shaft is adjusted to regulate air flow into the Masoala hall. Auxiliary flaps at the top of the roof and alongside the Masoala hall are closed. During particularly warm days, the ventilation shaft is fully open and auxiliary flaps are also opened to allow extra inflow of fresh air to cool down and dry off the air inside the Masoala hall. The dynamic regime and open regimes have similarities to the vented, dynamic flux chambers used for measuring gas exchange, only the Masoala hall is much larger.

During all operation regimes, the turbines are fully on with an air flow rate of 140’000 m^3^ h^–1^. During both the 2016 and 2017 measurement campaigns, all three operation regimes were used at different times. The maximal inflow rate of outdoor air was determined by wind speed measurements inside the ventilation shafts using cup as well as hot-wire anemometers. A pilot sampling campaign was made during 2016. Thus, the results presented in this paper focus on 2017.

### 2.3 Concentrations of Hg^0^, CO_2_, and CH_4_

Measurements were conducted between January and April 2016, and from March to April 2017. During these campaigns, Hg^0^ concentration was measured at the outflow of the Masoala hall (point A, Figure 1). Fresh air Hg^0^ concentrations were determined in January and February 2016 (point B, Figure 1), and during the 2017 campaign. Mixed inflow concentration measurements were performed from March–April 2016 (point C, Figure 1). Additionally, during the 2017 campaign, CO_2_ and CH_4_ concentrations were measured at locations A and B (Figure 1). We used a Tekran 1110 Two Port Sampling System (Tekran Instruments Corporation, Knoxville, TN, USA) to switch among the sampling lines. Turbulence data inside the Masoala hall were recorded by two sonic anemometers installed at two different heights on the visitor towers in April 2016 (Sect. [Sec sec2.4.4]).

Analysis of air Hg^0^ concentration was conducted using cold vapor atomic fluorescence spectroscopy (Tekran 2537X Automated Ambient Air Analyzer). The detection limit of the Tekran 2537X is lower than 0.1 ng m^–3^. The flow rate was 1.5 l min^–1^ and measurements were taken every 5 min from alternating cartridges. The instrument was automatically calibrated every 25 h. Additionally, manual calibrations of the permeation source were performed in the beginning and at the end of the campaigns using an external calibration device (Tekran 2505).

Mixing ratios of CO_2_ and CH_4_ (dry mole fractions) as well as water vapor (H_2_O) were measured with a cavity ring-down spectrometer (Picarro G2301, Picarro Inc., Santa Clara, California, USA), with a 24-h precision of ± 0.15 ppm of CO_2_ and ± 1 ppb CH_4_, respectively. The Tekran 2537 and the Picarro G2301 were connected to the Tekran 1110 sampling air from locations A, B and C in the ventilation system (Figure 1). Cycles of 10 min from each input source were used for the switching unit. This corresponds to two measurements of 5 min each for the Tekran 2537X.

### 2.4 Net Hg^0^ Flux Calculations

The rate of the Hg^0^ concentration change over time inside the hall was calculated according to eq 1 for the measurement campaign 2017.

$$\frac{\Delta c_{out}}{\Delta t}=\frac{q\cdot c_{\mathrm{inflow}}-q\cdot c_{outflow}+A\cdot F}{V}$$ 1

where Δc_out_ [ng m^–3^] is the difference between two measuring points of c_outflow_ over time step Δt, q [m^3^ h^–1^] is the air flow rate through the system, c_inflow_ and c_outflow_ [ng m^–3^] are the inflow and outflow Hg^0^ concentrations (measurement points B and A, see Figure 1), respectively, A [m^2^] is the area of the chamber, F is the net Hg^0^ flux [ng m^–2^ h^–1^] and V [m^3^] constitutes the volume of the chamber. The sections 2.4.1, 2.4.2 and 2.4.3 present equations to calculate the Hg^0^ flux depending on the operating regimes of the Masoala hall.

#### 2.4.1 Closed Regime: Nonvented Dynamic Flux Chamber

In the closed regime, the Masoala hall operates like a nonvented, dynamic flux chamber. The setup resembles the closed dynamic system applied to measure fluxes in floodplain ecosystems at the Elbe River.^56^ For the Hg^0^ concentration range observed in the Masoala hall, a linear description of the Hg^0^ concentration change is sufficient.

The net Hg^0^ flux (*F)* in ng m^–2^ h^–1^ results from eq 2

$$F=\frac{V}{A}\cdot \frac{\Delta c_{out}}{\Delta t}$$ 2

#### 2.4.2 Dynamic Regime: Standard-Vented, Dynamic Flux Chamber

In the dynamic regime, the Masoala hall operates like the dynamic flux chambers typically applied in many gas exchange studies.^38^ However, due to the large volume of the hall (200’000 m^3^) in relation to the rate that air is pumped into the glass house (140’000 m^3^ h^–1^), this standard-vented, dynamic flux chamber is not in a steady-state condition. Thus, the eq (eq 3) used to calculate the net Hg^0^ flux (*F*) in ng m^–2^ h^–1^ is

$$F=\frac{V}{A}\cdot \frac{\Delta c_{out}}{\Delta t}+q\cdot \frac{c_{outflow}-c_{\mathrm{inflow}}}{A}$$ 3

The first term on the right-hand side of the equation describes the accumulation of Hg^0^ (eq 2), while the second term describes the venting. In the second term, q [m^3^ h^–1^] describes the air flow rate through the system, and c_outflow_ and c_inflow_ [ng m^–3^] the outflow and inflow Hg^0^ concentrations, respectively. For our Hg^0^ flux calculation, time steps of Δt = 1 h were used. The ventilation generates a circular flow that was quantified at 140’000 m^3^ h^–1^ (Figure 1). The air flow through the open vent within the ventilation shaft was measured to contribute 41’000 m^3^ of fresh air per hour. Thus, in the dynamic regime, 29% of the total flow is fresh air. This results in a median fresh air turnover time of about 4.9 h.

#### 2.4.3 Open Regime: Vented, Dynamic Flux Chamber

In the open regime, the Masoala hall functioned like a vented, dynamic flux chamber. The Hg^0^ flux was calculated according to eq 3. The difference to the dynamic regime was that the total flow rate had to be newly defined because the auxiliary flaps along the hall were open. We determined the total flow rate based on net CH_4_ flux estimates (see details in Supporting Information S2). This approach was possible because we expected the net CH_4_ flux to be constant over the entire day. The reason for this is that CH_4_ concentrations are unaffected by stomatal activity and could therefore be used as a tracer. There is, however, uncertainty due to the insufficient data on the mixing of large amounts of incoming fresh air with the air inside the hall. We estimated the uncertainty of the CH_4_ emission flux as 8%, based on the IQR of the nightly CH_4_ emission flux measurements (see details in Supporting Information S1). The new flow rate q was calculated for each time step Δt = 1 h. During the open regime, the median fresh air flow rate was 143’000 m^3^ h^–1^ and increased up to 656’000 m^3^ h^–1^ due to the chimney effects inside the hall. The median fresh air turnover time decreases to 1.4 h compared to the dynamic regime.

#### 2.4.4 Micrometeorological Approach

The first step to determine the micrometeorological Hg^0^ flux and Hg^0^ dry deposition rates inside the Masoala hall was to obtain turbulence data (three-dimensional wind velocity fluctuations, speed of sound and/or virtual temperature derived from speed-of-sound measurements) at two heights from 15–28 April 2016. The measurements on the lower level (10.1 m a.g.l.) were conducted with a Gill R2A sonic anemometer (Gill Ltd., Lymington, UK). The sonic anemometer was attached to the guardrail of a bridge connecting the two visitor towers inside the Masoala hall. The measurements on the higher level were performed at 18.8 m a.g.l. using a Gill HS-100 sonic anemometer (Gill Ltd., Lymington, UK). This sonic anemometer was installed horizontally on top of the tallest visitor’s tower inside the Masoala hall. The Gill R2A sonic anemometer was mounted in a downward-looking manner at a 20° angle below the horizontal plane, and wind vector coordinates were rotated after data collection to match the coordinate system of the Gill HS-100. Turbulence data were recorded at 20 Hz (Gill HS-100) and 20.83 Hz (Gill R2A), respectively, using a Raspberry Pi embedded Linux computer system.

In a second step, we aggregated the raw data to 10 min average mean wind speed components (horizontal wind speeds *u̅* and *v̅*, and vertical wind speed *w̅*), virtual temperature $\left(\overset{®}{T_{v}}\right)$ , momentum flux $\left(\overset{®}{m^{\prime }w^{\prime }}=\sqrt{\overset{®}{u^{\prime }w^{\prime 2}\;}+\overset{®}{v^{\prime }w^{\prime 2}}}\right)$ , and sensible heat flux $\left(\overset{®}{w^{\prime }T_{v}^{\prime }}\right)$ . Here, the overlines denote time averaging, and primes short-term deviations from the temporal means. We then calculated the friction velocity u_*_ [m s^–1^] with eq 4 as follows:

$$u^{*}=\sqrt{\overset{®}{m^{\prime }w^{\prime }}}$$ 4

In a third step, we calculated the net Hg^0^ flux [ng m^–2^ h^–1^] based on the Hg^0^ concentration measurements (also available at 10 min resolution) and the modeled deposition velocity v_d_ [m h^–1^][Bibr ref57] using eq 5,

$$F_{Hg^{0}}=-v_{d}\cdot c_{Hg^{0}}$$ 5

The Hg^0^ concentration c_Hg0_ [ng m^–3^] above the vegetation was calculated with eq 6 as the mean of the concentration measurements made at inlet and outlet of the hall,

$$c_{{Hg}^{0}}=\frac{c_{\mathrm{inflow}}+c_{\mathrm{outflow}}}{2}$$ 6

This approach is typically used in concentration-monitoring networks, where it is known as the inferential method.^58^ A key assumption is that there is primarily Hg^0^ deposition and not Hg^0^ re-emission. Negative Hg^0^ fluxes indicate foliage Hg^0^ uptake, whereas positive fluxes denote Hg^0^ emission from the entire rainforest ecosystem into the Masoala hall’s air volume.

Deposition velocity was estimated using a resistance-based approach, considering three types of resistances in series:^59^ 1) the aerodynamic resistance (R_a_) describing the turbulent transport of Hg^0^ between air and surfaces, 2) the sublayer resistance (R_b_) reflecting the transfer of molecules across the laminar boundary layer around each leaf or other surfaces, and 3) a canopy resistance (R_c_) that has a high value at night when stomata are closed, but has a relatively low value during the day when plants are exposed to light, open their stomata and photosynthesize. We refer to R_c_ as canopy resistance rather than bulk surface resistance because the contribution of other resistances such as the resistance at the ground surface are small in dense vegetated canopies.

The deposition velocity v_d_ [m s^–1^] is calculated with eq 7 as follows:

$$v_{d}=\frac{1}{R_{a}+R_{b}+R_{c}}$$ 7

The aerodynamic resistance (R_a_) is quantified using the friction velocity u_*_ and mean horizontal wind speed $\mathrm{m̅}=\sqrt{\overset{®}{u^{2}}+\overset{®}{v^{2}}}$ , according to eq 8

$$R_{a}=\frac{\mathrm{m̅}}{{u_{*}}^{2}}$$ 8

The sublayer resistance (R_b_) was approximated using the empirical approach (eq 9) described by Thom^60^ for rather rough surfaces,^61^

$$R_{b}=6.2\cdot {u_{*}}^{-0.67}$$ 9

The canopy resistance (R_c_) is a combination of a relatively high cuticular resistance (dominant at night when stomata are closed) and a stomatal resistance that decreases curvilinearly as global radiation (Rg, in W m^–2^) increases.^62,63^ Here, eq 10 describes how R_c_ changes with global radiation (Rg), a standardized radiation response that is commonly used in many Jarvis type ET and SVAT models (e.g., Mosaic LSM^64^). Using a value of 400 W m^–2^ serves as a reference point where stomatal resistance effectively reaches a minimum under clear-sky conditions.

$$R_{c}=\{\begin{array}{ll} R_{c,\max}, & R_{g}=0\;{\mathrm{Wm}}^{-2}\\ R_{c,\min}+(R_{c,\max}-R_{c,\min})\cdot \left(1-{\frac{R_{g}}{400}}^{1/3}\right), & 0<R_{g}\leq 400\;{\mathrm{Wm}}^{-2}\\ R_{c,\min}, & R_{g}>400\;{\mathrm{Wm}}^{-2} \end{array}$$ 10

Following Wesely^59^ we use a high value of 9999 s m^–1^ for *R*_*c*, *max*_., which indicates that there is no stomatal Hg^0^ uptake and assume that $R_{c,\min}\approx \frac{1}{2}R_{c,\max}$ to calculate the initial Hg^0^ deposition velocity.

### 2.5 Calculating the Net CO_2_ Flux

The CO_2_ concentration change inside the Masoala hall was calculated for the measurement campaign 2017 as follows:

$$\frac{\Delta c_{out}}{\Delta t}=\frac{q\cdot c_{\mathrm{inflow}}-q\cdot c_{\mathrm{outflow}}+A\cdot U+A\cdot Q}{V}$$ 11

The difference between eq 1 and eq 11 is that we replaced the term for net Hg^0^ flux (A·F) in eq 1 with the terms (A·U + A·Q) to account for the net CO_2_ flux, composed by the gross CO_2_ uptake U by the vegetation [μmol m^–2^ s^–1^] and by the CO_2_ emission flux Q [μmol m^–2^ s^–1^], originating from respiration by soil, vegetation, animals and humans. In any regime, Q can be directly derived as described in eq 2, applied to nighttime conditions. The net CO_2_ flux inside the hall was then derived according to the Hg^0^ flux in the open regime (eq 3). In the closed regime, there is no influx of fresh air, thus, q and U are assumed to be zero.

### 2.6 Estimating the CO_2_ Uptake

The CO_2_ uptake (i.e., gross primary production; GPP) is the sum of the net CO_2_ flux (i.e., net ecosystem exchange; NEE) and total ecosystem respiration (R_eco_). Total ecosystem respiration was calculated using the net CO_2_ flux measured during nighttime, under the assumption that there was no photosynthesis (despite the presence of some epiphytes with CAM photosynthesis, albeit with little biomass compared to nonepiphytes) and that variation in air temperature inside the Masoala hall between day and night was in a similar range (daytime span: 17.8–25.4 °C; nighttime span: 18.4–24.5 °C).

### 2.7 Auxiliary Variables

Global radiation (Rg) was recorded with a CM21 pyranometer (Kipp & Zonen USA Inc., Bohemia, NY, USA) at the MeteoSwiss Zurich/Fluntern measurement station (47°22′41.56″ N, 8°33′56.37″ E; 579 m a.s.l.) about 1.25 km southwest of the Masoala hall. Air temperature (T_a_) inside the Masoala hall was determined using a CS215 Digital Air Temperature and Relative Humidity Sensor mounted with a 10-plate shield (Campbell Scientific Inc., Logan, UT, USA) at about 1 m above ground. The CO_2_ concentration data from the ICOS Jungfraujoch station were downloaded from the ICOS Carbon portal.^65^

## 3 Results

### 3.1 Hg^0^ and CO_2_ Concentrations

During the 2017 campaign, the median Hg^0^ concentration in the inflow air of the Masoala hall (position B; Figure 1) was 1.35 ng m^–3^ (Interquartile Range IQR, i.e., Q_0.25_ – Q_0.75_: 1.27–1.45 ng m^–3^). The median outflow Hg^0^ concentration was 1.04 ng m^–3^ (IQR: 0.95–1.11 ng m^–3^; position A; Figure 1). The outflow Hg^0^ concentrations were only 77% of the inflow concentrations, indicating rainforest Hg^0^ uptake (Figure 2a).

**Figure 2:**
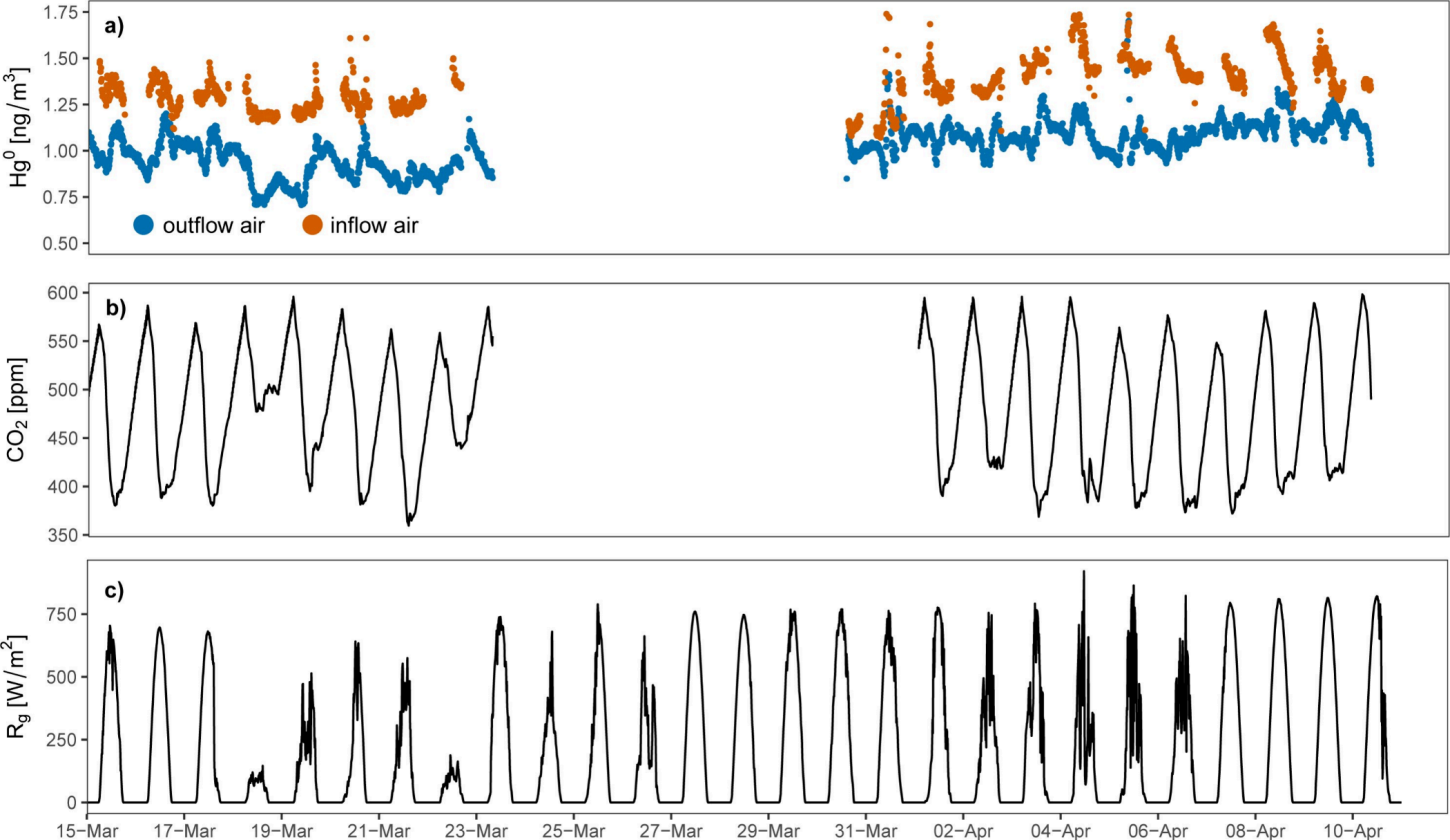
Time series of Hg^0^ and CO_2_ concentrations as well as measured global radiation (14 March–10 April, 2017). (a) Air Hg^0^ concentration in the outflow (blue dots, position A; Figure 1) and inflow (orange dots, position B; Figure 1) of the Masoala hall. (b) CO_2_ concentrations in the outflow of the Masoala hall (A in Figure 1). (c) Global radiation (Rg) measured outside the Masoala hall, at the Zurich/Fluntern (SMA) measuring station run by MeteoSwiss. Visitors were allowed in the Masoala hall between 10 a.m. and 6 p.m.

The concurrently measured median inflow CO_2_ concentration was 424 ppm (IQR: 416–436 ppm), 3% higher compared to CO_2_ concentrations of 411 ppm measured on the Jungfraujoch (3572 m a.s.l.) during the same period.^65^ The median outflow CO_2_ concentration was 472 ppm (IQR: 418–524 ppm). The outflow CO_2_ concentrations (Figure 2b) revealed distinct diel dynamics, with maximum concentrations around 5 a.m. and minimum concentrations around 1 p.m. when global radiation outside the Masoala hall peaked (Figure 2c). The minimum CO_2_ concentration of 375 ppm was measured on 21 March 2017 and the maximum concentration of 593 ppm on 10 April 2017 (Figure 2b). The steady decrease in CO_2_ concentrations during the day indicated photosynthetic CO_2_ assimilation by the rainforest, despite ongoing respiration of plants, soils, animals and visitors. The steady increase in CO_2_ concentrations during the night after the visitors were gone (after 6 p.m.) indicated CO_2_ emissions driven by total ecosystem respiration, i.e., from plants, soils, and animals.

### 3.2 Net Hg^0^ and CO_2_ Fluxes

The net median Hg^0^ flux was −0.94 ng m^–2^ h^–1^ (IQR: −2.22 – −0.24 ng m^–2^ h^–1^) and followed a clear diel pattern (Figure 3a), indicating weak Hg^0^ uptake during the night (Rg < 5 W m^–2^) and strong Hg^0^ uptake during the day (Rg ≥ 5 W m^–2^). During the night, the median net Hg^0^ flux was −0.24 ng m^–2^ h^–1^ (IQR: −0.44 – −0.01 ng m^–2^ h^–1^). Nighttime Hg^0^ uptake of −0.45 ng m^–2^ h^–1^ (IQR: −0.57 – −0.30 ng m^–2^ h^–1^) was calculated for 24 out of 63 nights during the closed regime. During these nights, we observed distinct nighttime Hg^0^ depletion (R^2^ > 0.8, *p* < 0.01; e.g., in the night from 14–15 March 2017; Supporting Information S3). During the day, the median net Hg^0^ flux was −2.3 ng m^–2^ h^–1^ (IQR: −3.45 – −1.69 ng m^–2^ h^–1^). The net Hg^0^ uptake reached a maximum between 10 a.m. and 14 p.m. and peaked at −3.71 ng m^–2^ h^–1^. The flux of Hg^0^ taken up by the forest during the day correlated with global radiation (R^2^ = 0.3, *p* < 0.01; Supporting Information S4). Thus, Hg^0^ uptake was −2.64 ng m^–2^ h^–1^ during sunny days (≥347 W m^–2^) and −2.00 ng m^–2^ h^–1^ during cloudy days (<347 W m^–2^). Overall, midday Hg^0^ uptake exceeded nighttime Hg^0^ uptake by almost a factor of 10.

**Figure 3:**
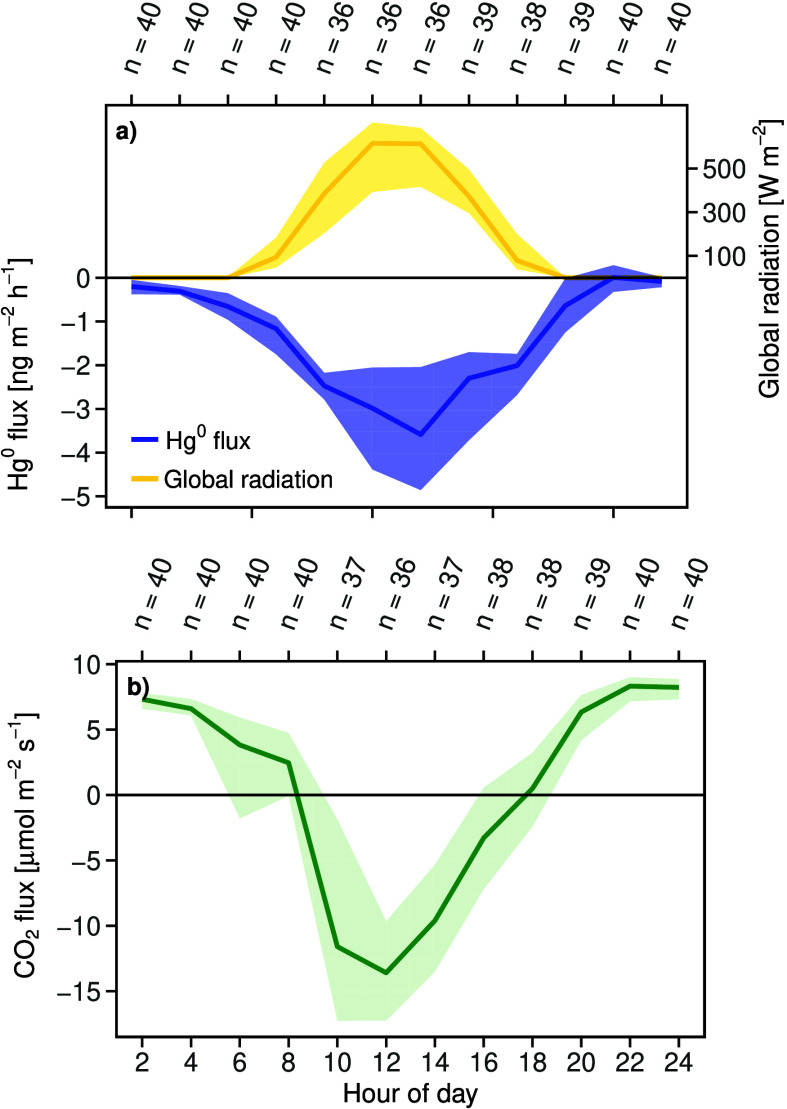
Diel cycles of global radiation, net Hg^0^ and CO_2_ fluxes inside the Masoala hall. (a) Two hourly aggregated global radiation and net Hg^0^ flux, (b) concurrently measured, 2 hourly aggregated net CO_2_ flux obtained from 14 March to 10 April, 2017. The lines indicate median values, and the bands represent the interquartile ranges (Q_0.25_ to Q_0.75_). For each 2 h aggregate, between 36 and 40 observations have been made. Visitors are allowed in the Masoala hall between 10 a.m. and 6 p.m.

The net CO_2_ flux (NEE) indicated total ecosystem respiration (R_eco_) dominating during the night (6 p.m. to 8 a.m.) and CO_2_ uptake (GPP) dominating during the day (8 a.m. to 6 p.m.; Figure 3b). The median net CO_2_ flux was 3.94 μmol m^–2^ s^–1^ (IQR: −4.57–7.33 μmol m^–2^ s^–1^), indicating substantial nighttime respiration from plants, soil and animals. The amount of CO_2_ fixed by the rainforest through photosynthesis increased very fast in the morning from about 7 to 9 a.m. (Figure 3b), and reached peak values around 11 a.m. They were followed by an almost linear decrease in NEE until 7 p.m. Hence, the diel cycle of the net Hg^0^ flux was strongly correlated to the diel cycle of GPP (R^2^ = 0.89, *p* < 0.01, Supporting Information S5).

### 3.3 Cumulative Net Hg^0^ Flux and GPP

The cumulative Hg^0^ flux and cumulative GPP calculated from 14 March – 10 April 2017 was −0.65 μg Hg^0^ m^–2^ and −137 g C m^–2^, respectively (Figure 4). This results in a daily uptake rate of 33.5 ng Hg^0^ m^–2^ and 7.0 g C m^–2^. Temporal upscaling of the available data to one year resulted in an annual rainforest Hg^0^ uptake of 12.2 ± 0.5 μg Hg^0^ m^–2^ and a GPP of 2570 ± 126 g C m^–2^ (mean ± SE, based on daily variability). These estimates assume that the observation period was representative of annual conditions. The median daytime global radiation penetrating through the roof of the Masoala hall was 70% of the Rg measured outside the hall (260 W m^–2^) and was lower compared to typical average daytime global radiation in the tropics of 350 W m^–2^.^66^ Given the linear relationship between photosynthetically active radiation (PAR) and Rg^67,68^ as well as PAR and GPP (if light use efficiency remains constant and no environmental stresses from air temperature or water availability occur) a 30% elevated photosynthetic activity in the tropics would lead to a proportional increase in GPP.^69,70^ Thus, the estimated annual Hg^0^ and CO_2_ uptake of the Masoala Rainforest was 17.5 μg Hg^0^ m^–2^ and 3672 g C m^–2^.

**Figure 4:**
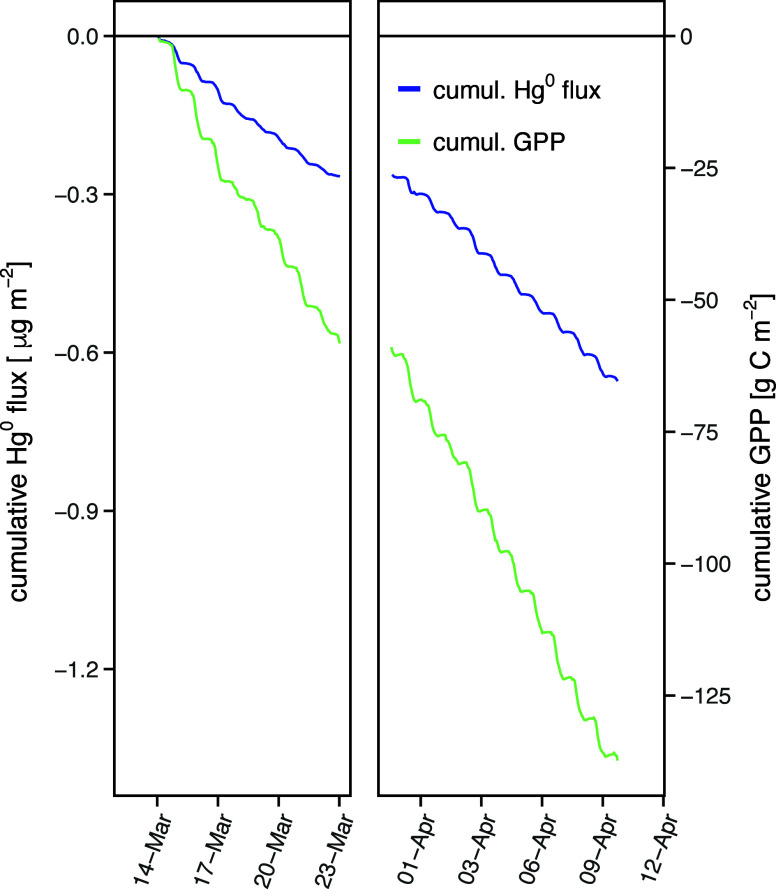
Cumulative fluxes of the net Hg^0^ flux and CO_2_ flux inside the Masoala hall. The aggregated fluxes were calculated for the period from 14 March to 10 April, 2017.

### 3.4 Evidence for Stomatal Hg^0^ Uptake

The unique setting of concurrent dynamic flux chamber and micrometeorological measurements within the Masoala hall allowed us to derive a rainforest canopy resistance (R_c,min_). We found that using an arbitrarily chosen R_c,min_ = 1/2 R_c,max_, indicating very limited stomatal Hg^0^ uptake, the median daytime Hg^0^ deposition velocity of 0.02 cm s^–1^ would be too low (Figure S6a in the Supporting Information) and the median Hg^0^ flux would not follow the same diel cycle compared to the dynamic flux chamber approach (Figure S6b in the Supporting Information). To align the Hg^0^ fluxes derived from micrometeorological measurements to the net flux patterns of the glasshouse chamber, we parametrized the R_c,min_. Using an R_c,min_ of 1000 s m^–1^, i.e. $\frac{1}{10}R_{c,\max}$ the diurnal Hg^0^ uptake from the micrometeorological approach matched that arrived at when treating the Masoala hall as a dynamic flux chamber, with Hg^0^ uptake of 3.7 ng m^–2^ h^–1^ around noon (Figure 5b). The newly derived median daytime rainforest Hg^0^ deposition velocity increased to 0.04 cm s^–1^ (Figure 5a) The overall median Hg^0^ flux was −0.66 ng m^–2^ h^–1^ (IQR: −1.77 – −0.40 ng m^–2^ h^–1^) and the daytime Hg^0^ flux was −1.77 ng m^–2^ h^–1^ (IQR: −3.25 – −1.0 ng m^–2^ h^–1^) (Figure 5b). To the best of our knowledge, we present the first attempt to estimate a canopy resistance for Hg^0^ for a rainforest ecosystem based on ecosystem-scale flux methods.

**Figure 5:**
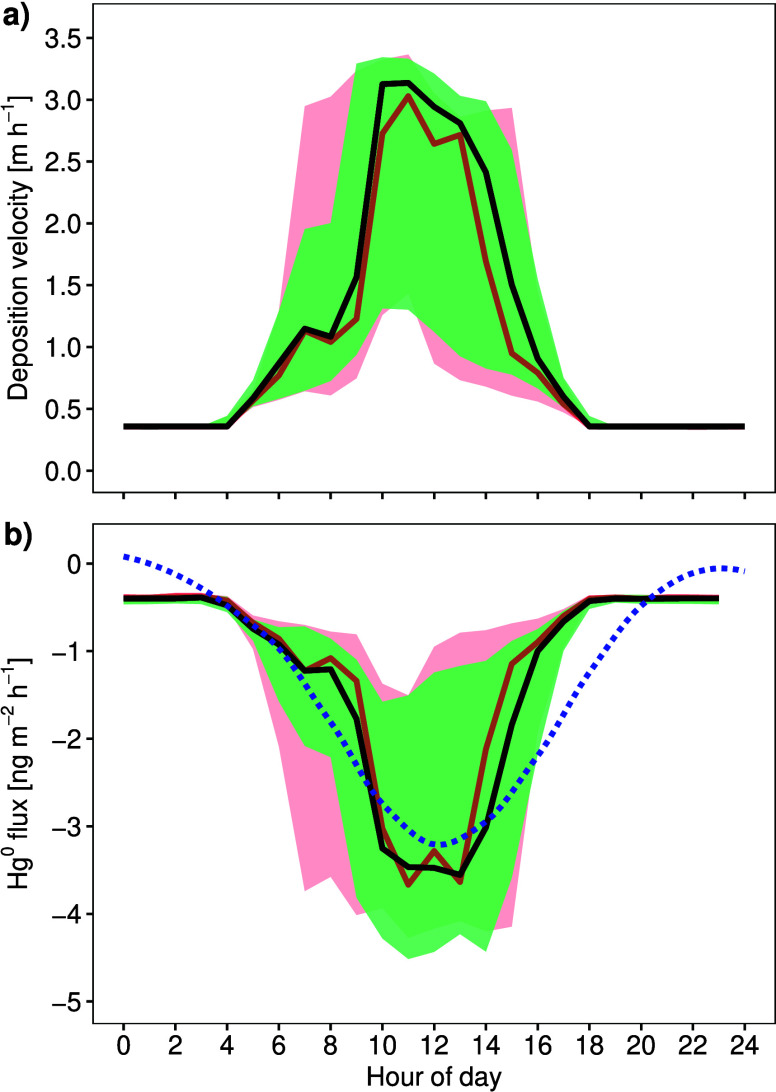
Diel pattern of the Hg^0^ deposition velocity and Hg^0^ flux inside the Masoala hall based on micrometeorological determination of turbulence. The Hg^0^ deposition velocity (a) and Hg^0^ flux (b) based on the calibration of R_c_ where R_c,min_ = $\frac{1}{10}$ R_c,max_. Black lines with green areas show data obtained from the sonic anemometer deployed at 18.8 m height, and red lines with red areas show data from 10.1 m height. Colored areas show the interquartile ranges (Q_0.25_ to Q_0.75_). The data were obtained from April 15 to 28, 2016. The blue smoothed, dashed line represents the 2 hourly medians of the net Hg^0^ flux measured using the dynamic flux chamber approach (presented in Figure 3a).

## 4 Discussion

### 4.1 Rainforest Net Hg^0^ Flux Driven by Stomatal Hg^0^ Uptake

Net rainforest Hg^0^ fluxes measured inside the Masoala hall revealed a diel pattern of continuous Hg^0^ uptake that identifies the rainforest as an atmospheric Hg^0^ sink. A major driver of the Hg^0^ flux was stomatal uptake related to photosynthetic CO_2_ assimilation (GPP). This is reflected by the significant negative linear relationship between the net Hg^0^ flux and global radiation (R^2^ = 0.30, *p* < 0.01) as well as in the significant positive relationship between the cumulative Hg^0^ flux, i.e. Hg^0^ uptake and cumulative GPP, i.e. CO_2_ uptake (R^2^ = 0.89, *p* < 0.01). The diel Hg^0^ pattern conflicts with the often reported positive correlations between global radiation and terrestrial Hg^0^ fluxes from soils back to the atmosphere driven by photoreduction of Hg^II^ on surfaces followed by Hg^0^ re-emission.^39^ Instead, we found net Hg^0^ flux into the forest correlated with global radiation (Figures 3, 5). Due to the small span of air temperatures measured at ground level inside the hall (IQR: 19.4–23.3 °C), there was no effect of air temperature change on the Hg^0^ flux (R^2^ < 0.1, *p* > 0.01). During the entire campaign, neither air temperature nor humidity (i.e., wet air mole fraction; measured with the Picarro G2301) had an influence on net Hg^0^ flux.

The key role of stomatal uptake in driving the net Hg^0^ flux into the Masoala Rainforest is supported by two features of the observations. One is that Hg^0^ uptake exhibited a diel variation similar to that of GPP, with a peak between 10 a.m. and 2 p.m., when leaf diffusive conductance reaches a maximum (Figure 3).^71^ Second, without accounting for stomatal Hg^0^ uptake, net Hg^0^ uptake derived from micrometeorological measurements would be too low (Figure 5b vs Figure S6b in the Supporting Information).

The deposition velocity of the Masoala Rainforest fell within the top 25% of forest Hg^0^ dry deposition velocities parametrized by the GEOS-Chem BASE model (IQR: 0.035- 0.039 cm s^–1^).^6^ The determined Hg^0^ dry deposition velocity was slightly higher compared to Hg^0^ dry deposition velocities from flux bag observations in deciduous forests excluding dry seasons in the subtropical areas (0.01–0.03 cm s^–1^),^14,22,29,30^ and overlapped with those based on litterfall (0.020–0.041 cm s^–1^) and total foliar uptake (0.030–0.055 cm s^–1^) observations mainly performed in temperate and boreal forests in the northern Hemisphere.^46^ The peak daytime Hg^0^ dry deposition velocities (0.08 cm s^–1^, Figure 5a) were similar to Hg^0^ uptake rates measured in a midlatitude broadleaf forest (0.071 cm s^–1^) using micrometeorological techniques.^36^ These Hg^0^ uptake rates in the Masoala Rainforest, however, were still much lower compared to deposition velocities derived from litterfall (0.17 cm s^–1^) and total foliar uptake (0.35 cm s^–1^) in the Amazon rainforest.^46,72^ Remarkably, the observed nighttime Hg^0^ flux indicated that Hg^0^ uptake by vegetation also occurred at night (Figures 3, 5, S3). This has been observed by above canopy net Hg^0^ flux measurements for a subtropical forest,^37^ but contrasts with diel Hg^0^ flux patterns in midlatitude broadleaf forest,^36^ where nighttime Hg^0^ re-emission was observed. Thus, our net Hg^0^ flux measurements implied that tropical forests take up Hg^0^ not only during the day, but also during the night at rates exceeding nighttime Hg^0^ re-emission, albeit at much lower rates than during daytime.

Re-emission of Hg^0^ was never dominant over Hg^0^ uptake in the Masoala Rainforest. However, the absence of net Hg^0^ re-emission does not necessarily indicate low Hg^0^ re-emission rates. The overall positive net CO_2_ flux, i.e., total ecosystem respiration, measured at night is likely to be associated with substantial soil Hg^0^ re-emission. That is because microbial decay of organic matter promotes Hg^0^ re-emission from soils.^73^ Nonstomatal Hg^0^ uptake is likely to contribute to the net Hg^0^ uptake even at night, and helps to counterbalance re-emission from the soils. The possibility that stomata remain open during the night in rainforests with good access to water may also contribute to some net Hg^0^ uptake at night.

### 4.2 Rainforest Hg^0^ Uptake: Weaker Hg^0^ Sinks than Midlatitude Broadleaf Forests?

The estimated annual Hg^0^ uptake of the Masoala Rainforest (17.5 μg m^–2^ yr^–1^) was higher compared to the Hg^0^ uptake of a tropical rainforest in China (12.6 μg m^–2^ yr^–1^) estimated using stable Hg isotope analyses.^74^ Still, these two net rainforest Hg^0^ fluxes were lower compared to the net Hg^0^ uptake by a subtropical forest of 53.9 ± 19.8 μg m^–2^ yr^–1^ ^37^ and a midlatitude broadleaf forest of 25.1 ± 2.4 μg m^–2^ yr^–1^.^36^ The differences in the net Hg^0^ uptake between the rainforests and the other broadleaf forests can be explained by differences in the total input fluxes, i.e., stomatal Hg^0^ uptake that is reflected by GPP,^31^ nonstomatal uptake, and output fluxes, i.e., Hg^0^ re-emission from the rainforest floor.^74^

The estimated annual GPP of the Masoala Rainforest (3672 g C m^–2^) falls within the range of the annual mean GPP in a tropical rainforest in French Guiana (annual average GPP varied from 3385–4061 g C m^–2^)^75^ and in the tropical rainforest region as a whole (3551 ± 160 g C m^–2^).^76^ The GPP typically decreases with latitude and is higher in the tropical forests compared to midlatitude broadleaf forests.^77^ As examples of the midlatitude broad leaf forests, GPP was 1441 and 1526 g C m^–2^ yr^–1^ at the Harvard Forest, USA^78^ and 1830 g C m^–2^ yr^–1^ at the Lägeren forest in Switzerland.^79^ Since Hg^0^ uptake is closely related to the stomatal conductance and, therefore, to the photosynthetic activity of the plants,^25^ we can assume higher Hg^0^ uptake rates in the tropics compared to midlatitude forest. This is reflected by elevated rainforest foliage uptake and litterfall, which are about 1.5 times^13^ and more than two times^45^ higher compared to midlatitude forests, respectively.

The net ecosystem Hg/C uptake ratio of the Masoala Rainforest was 0.05 μg Hg g^–1^ C. This is lower than the ratio measured at a subtropical broadleaf forest (0.067 μg g^–1^) or a midlatitude broadleaf forest (0.091 μg g^–1^) during the growing season.^31^ These lower Hg/C uptake ratios, and the lower net Hg^0^ uptake in our measurements at Masoala despite higher GPP could result from higher emission of Hg from the soils. However, we hesitate to suggest that higher soil evasion is a factor in tropical forests that reduces the net Hg^0^ flux, for three reasons. First, we have not measured net exchange between the soil and the atmosphere below the forest canopy inside the Masoala hall. Second, soil Hg^0^ re-emission from the artificially constructed soils might be different compared to natural rainforest soil, even though the soil layers were composed to be similar to those on Madagascar. Third, respiration by visitors likely increases total ecosystem respiration from 10 a.m. to 6 p.m. We estimated a human respiration rate of 1.08 g C m^–2^ day^–1^ and concluded that we might underestimate GPP by about 15% (see details of this calculation in Supporting Information S1). To further investigate the relationship between CO_2_ and Hg^0^ uptake in rainforests, the processes of soil respiration and soil Hg^0^ re-emission at day and night should be investigated, as well as Hg^0^ exchange between the soils and the air beneath the forest canopy.^13,74^

### 4.3 Implications

Tropical and subtropical forests account for about 30% of the global forest net carbon uptake of 7.6 Gt CO_2_ yr^–1^.^80^ Typically, rainforests are considered sinks for CO_2_ but ecosystem-specific differences in the net CO_2_ flux span from net sources (375 ± 179 g C m^–2^ yr^–1^) at a site on Kalimantan, Indonesia to net sinks (−1190 ± 172 g C m^–2^ yr^–1^) at a site in Brazil.^81^ Since stomatal Hg^0^ uptake correlates with CO_2_ assimilation, rainforests are typically considered terrestrial sinks for atmospheric Hg^0^ as well.^13^ Tropical moist broadleaf forests (27.3 μg m^–2^) and tropical dry broadleaf forests (24.6 μg m^–2^) have been identified as the strongest sinks for Hg^0^ among the major global biomes, while temperate deciduous/mixed and coniferous forests take up 18.3 and 14.3 μg Hg^0^ m^–2^ every year, respectively.^13^ The net Hg^0^ uptake of the Masoala Rainforest and the Xishuangbanna Rainforest^74^ was lower than the predicted rainforest net Hg^0^ uptake^13^ and what was indicated by total Hg measurements in foliage and litterfall.^16,44^ However, our derived rainforest Hg^0^ deposition velocities were in line with the forest Hg^0^ deposition velocities parametrized by the GEOS-Chem BASE model.^6^ The indication from this study of relatively low net Hg^0^ uptake rates from rainforests would further reduce the newly estimated global flux of Hg^0^ dry deposition to land (2276 Mg yr^–1^).^46^

Based on the Masoala Rainforest results, we conclude that canopy Hg^0^ uptake was a dominant Hg^0^ deposition process in rainforests. Since there is no plant physiological parametrization of Hg^0^ uptake, we advocate for more experiments to confirm the R_c,min_ for rainforests given the importance of this parameter for global models of Hg exchange between the atmosphere and earth surface. Finally, to better constrain the forest Hg^0^ sink strength and improve Hg^0^ flux process understanding, we also recommend more ecosystem-specific net Hg^0^ exchange measurements,^82^ ideally colocated with CO_2_ eddy covariance systems, and partitioning of the net Hg^0^ flux components using stable Hg isotope techniques. This should include the identification of stomatal and nonstomatal Hg^0^ uptake processes as well as soil re-emission.

## Supplementary Material



## Data Availability

The original data that support the findings of the study are available at ETH Zurich’s Research Collection at https://doi.org/10.3929/ethz-c-000782612.

## Notes

Additional information on the Masoala hall and methodological uncertainties (section S1), concentration and flux estimates of CH_4_ (section S2), depletion of Hg^0^ during the night (section S3), correlation between global radiation and Hg^0^ uptake (section S4), correlation between Hg^0^ flux and CO_2_ uptake (section S5), and deposition modeling based on a different R_c_ (section S6) (PDF)
