# Supplementary material for: Uptake of Gaseous Elemental Mercury by a Rainforest: Insights from a Tropical Glasshouse Used as a Dynamic Flux Chamber
Source: Environ Sci Technol. 2025 Aug 26;59(35):18675–86. doi: 10.1021/acs.est.5c05823 (PMC12424189; doi:10.1021/acs.est.5c05823)
Supplement: Supplementary file 1 [file es5c05823_si_001.pdf]

Supporting Information for

Uptake of gaseous elemental mercury by a rainforest: Insights from a tropical glasshouse used as a dynamic flux chamber

*Basil Denzler [a,b], Werner Eugster [c♦], Christian Bogdal [a,d], Kevin Bishop [e], Nina Buchmann [c], Konrad Hungerbühler [a] and Stefan Osterwalder [c,f]**

[a]Institute for Chemical and Bioengineering, Department of Chemistry and Applied Biosciences, ETH Zurich, 8093 Zurich, Switzerland

[b]Kantonsschule im Lee, 8400 Winterthur, Switzerland

[c]Institute of Agricultural Sciences, Department of Environmental System Sciences, ETH Zurich, 8092 Zurich, Switzerland

[d]Zurich Forensic Science Institute, 8004 Zurich, Switzerland

[e]Department of Aquatic Sciences and Assessment, Swedish University of Agricultural Sciences, 75007 Uppsala, Sweden

[f]Hydrology and Climate, Department of Geography, University of Zurich, 8057 Zurich, Switzerland

* Email: stefan.osterwalder@geo.uzh.ch

♦ W.E. Deceased on May 23, 2022

This supporting information contains: 8 pages including 10 figures.

Contents

S1: The Masoala hall – a large dynamic flux chamber S2

S2: Concentration and flux estimates of CH_4 .... S4

S3: Depletion of Hg^0 during the night S5

S4: Global radiation drives Hg^0 uptake S5

S5: Strong relationship in diel variation between Hg^0 flux and CO_2 uptake S6

S6: Deposition velocity based on a different Rc S7

References S8

S1: The Masoala hall – a large dynamic flux chamber

Dynamic flux chambers (DFCs) are the most frequently applied techniques to measure the Hg^0 flux (85% of all flux measurements).[1] DFCs are typically much smaller (0.03 m^3) than the Masoala hall (200’000 m^3). Up to now, the largest DFCs used to study Hg^0 fluxes had a capacity of 180 m^3.[2-4] Hence, our study site was about 1000 times larger (Fig. S1a). Here, we measured the Hg^0 concentration using active Tekran 2537X instruments (Fig. S1b at the DFC inlet [Fig. S1c] and the DFC outlet [Fig. S1d]). The calculated turnover time was 4.9 h for the dynamic regime. Compared to previous DFC studies,[5,6] this is about 30 times lower. Compared to other DFCs, an important advantage was that turbulence inside the Masoala hall did not fluctuate over time. That allowed for a direct comparison of Hg^0 fluxes derived with the DFC and the micrometeorological method. We had no issues regarding an adjustment of the flow rate to set an optimal turnover time.[7]

Methodological uncertainties

Possible sources of uncertainties in our setup lie in the construction of the hall (not made of Teflon), that we were not able to perform blank measurements and that the hall was open to visitors from 10 a.m. to 6 p.m. The ventilation tubing, the concrete of the foundation, and the heat exchange elements in the ventilation system could all constitute possible sinks or sources of Hg^0. However, the Masoala hall has already been in operation since 2003, i.e., 13 years before our measurement campaign started. Because surface effects decrease with an increasing DFC volume (due to the surface volume ratio), it is very likely that an equilibrium between hypothetical Hg^0 sinks and sources has been established. We feel encouraged in this assumption because we did not observe any changes in Hg^0 concentration during a heating period, when the heating elements were operating. Furthermore, such surface effects would not be able to explain the variability in Hg^0 uptake observed over the diel cycle. We are therefore confident that we indeed observed Hg^0 uptake by the rainforest. Using error propagation, we estimated the uncertainty range for the 656,000 m^3/h flow rate to be 34%. Therefore a 30% uncertainty for the exchange flux was assumed, which was estimated by the difference between $c_{outflow}$ and c_{inflow} in our model. The uncertainty of Δc was estimated at 5%, and the uncertainty of the CH_4 emission flux was estimated at 8%, based on the IQR of the nightly CH_4 emission flux measurements.

While there is no evidence that humans with dental amalgam fillings increase Hg^0 concentrations in such large and mostly ventilated rooms, it is likely, however, that visitors, exhaling CO_2 as a byproduct of respiration, contributed to the daytime CO_2 concentration

measured inside the Masoala hall. If there were 1.18 million visitors distributed evenly over the course of the year[8] and one third of them visited the Masoala hall (this is an estimate because the number of visitors entering the Masoala hall was not determined), there were 393’721 visitors in 2016. This equates to approximately 1078 visitors per day. With the facility open for eight hours per day, this translates to an average of 134 visitors per hour. Per visitor we assume an average residence time of one hour inside the hall and thus about 40 grams of CO_2 emissions.[9] Since the area of the facility is 10’856 m^{-2}, the C emission rate is 0.14 g m^{-2} h^{-1}. Thus, the total human C emissions accounted for about 1.08 g m^{-2} per eight-hour day. Additionally, animals present at the facility contributed to total ecosystem respiration, which is accounted for in the measurements.

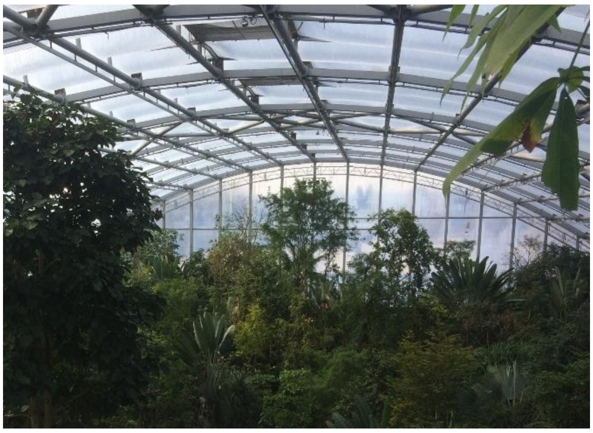

Figure S1a. View from one of the visitor towers inside the Masoala Rainforest hall.

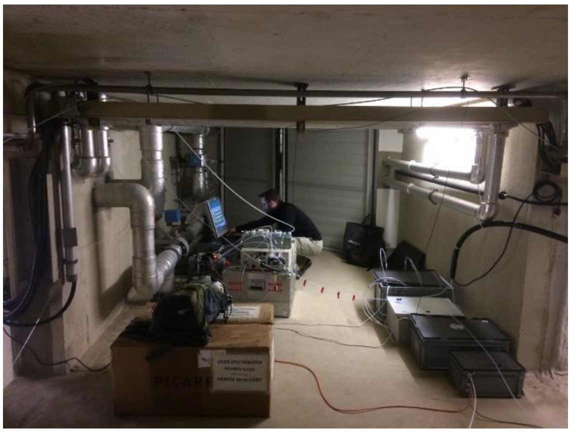

Figure S1b. Instrumentation to measure Hg^0, CO_2 and CH_4 concentrations at the inflow and outflow of the Masoala Rainforest hall.

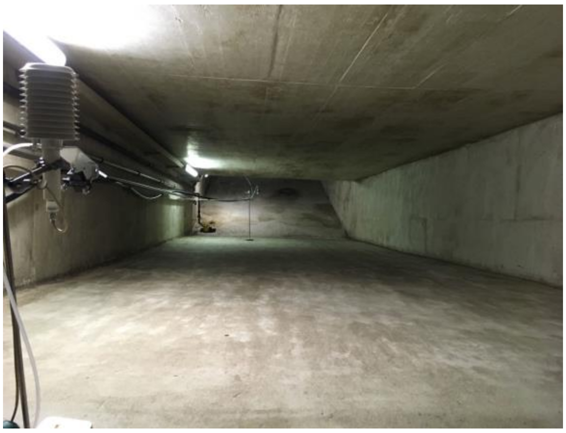

Figure S1c. Location of the sample inlet to measure dynamic flux chamber inflow air Hg^0 concentrations.

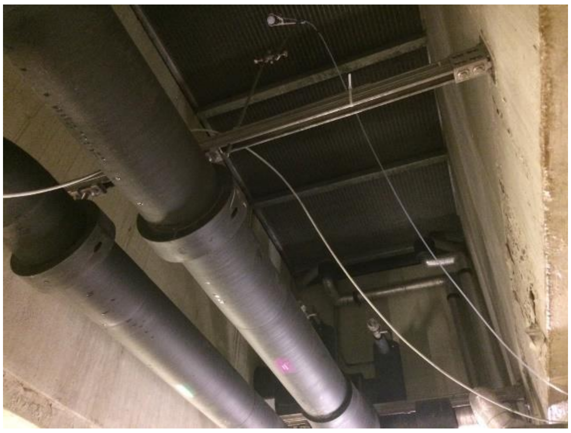

Figure S1d. Location of the sample inlet to measure dynamic flux chamber outflow air Hg^0 concentrations.

S2: Concentration and flux estimates of CH_4

The net CH_4 flux was derived from the steady increase of CH_4 concentrations during the night (linear model, $r^2 > 0.9$). The median net CH_4 flux was very low, 0.018 μmol m^{-2} s^{-1} (IQR: 0.016 - 0.022 μmol m^{-2} s^{-1}) and remained very stable throughout March–April 2017.

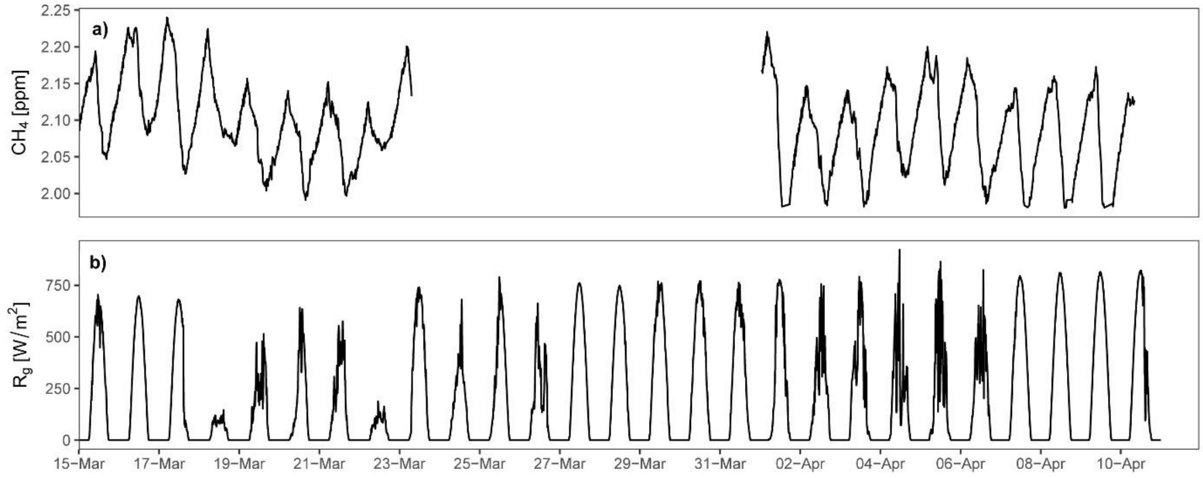

Figure S2a. Time series of CH_4 concentrations and global radiation (Rg) measured inside the Masoala hall. The CH_4 concentrations (a) and Rg (b) were recorded from 15 March–10 April 2017. The CH_4 concentrations generally increased during the night and decreased during the day. The decrease in CH_4 during the day was less pronounced when global radiation was weak.

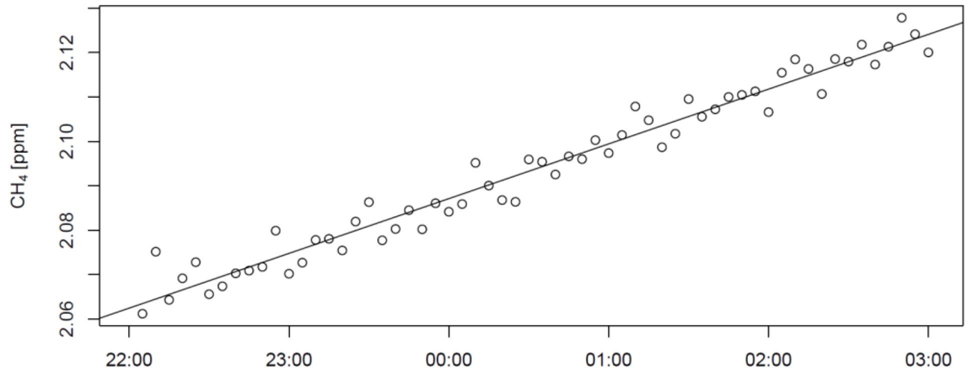

Figure S2b. Increase of CH_4 concentrations during the closed regime at night. An exemplary slope of the CH_4 concentration increased during the nights from 15–22 March, 2017.

S3: Depletion of Hg^0 during the night

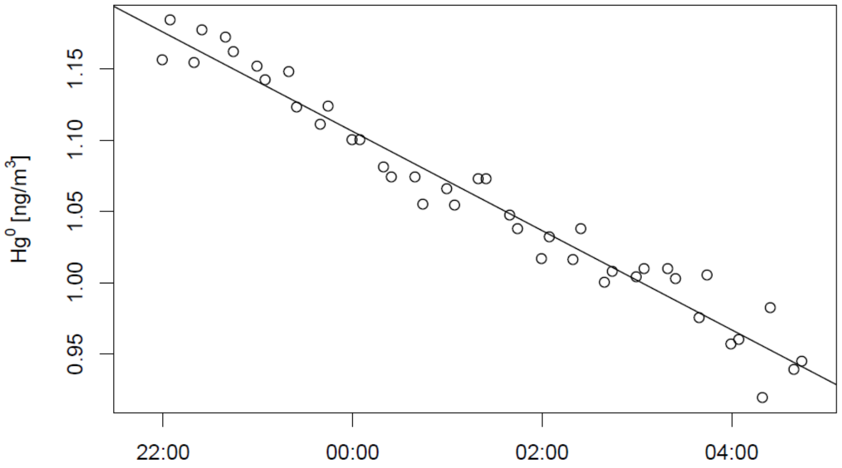

Figure S3. Depletion of Hg^0 concentrations inside the Masoala hall during the closed regime. An exemplary slope of the Hg^0 concentration decrease over time is shown for the night from 14–15 March, 2017. Based on the 42 observations, a deposition flux of -0.63 ng m^{-2} h^{-1} with an R^2 of 0.96 was obtained.

S4: Global radiation drives Hg^0 uptake

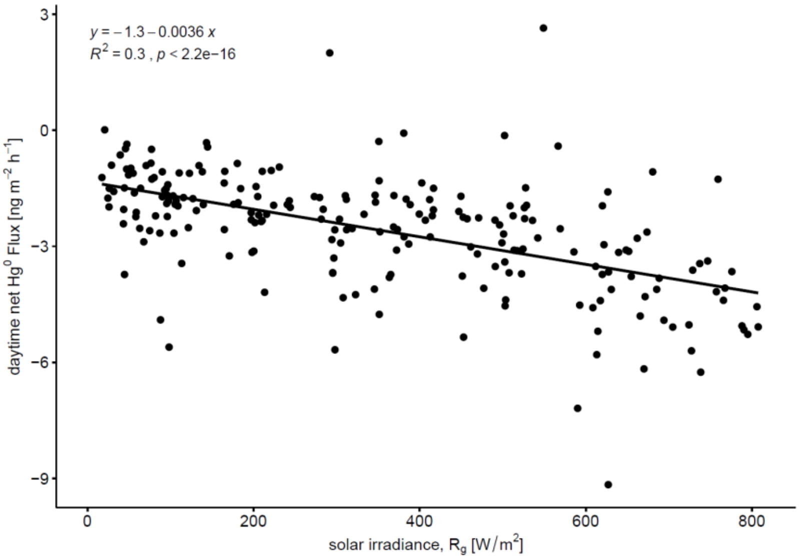

Figure S4. Linear correlation between global radiation and the net Hg^0 flux during the day.

S5: Strong relationship in diel variation between Hg^0 flux and CO_2 uptake

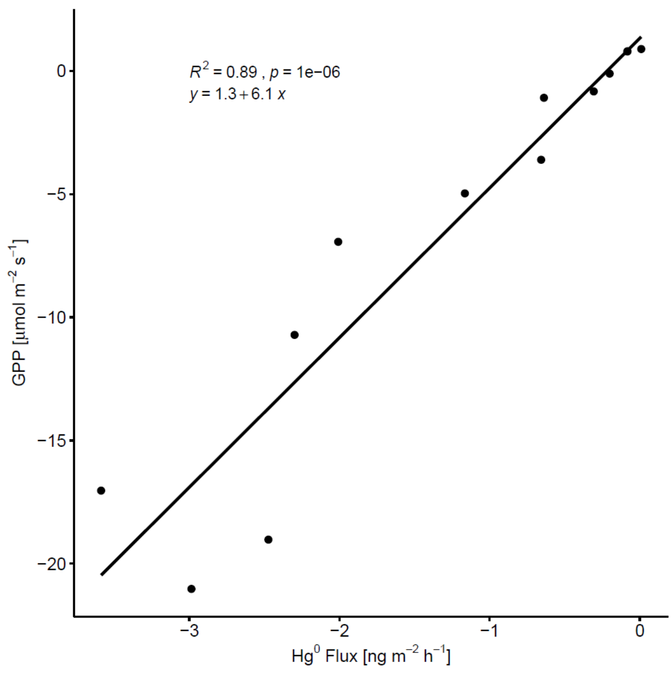

Figure S5. Linear correlation between the 2-hourly median values of the net Hg^0 flux and GPP, measured from 14 March–10 April 2017.

S6: Deposition velocity based on a different Rc

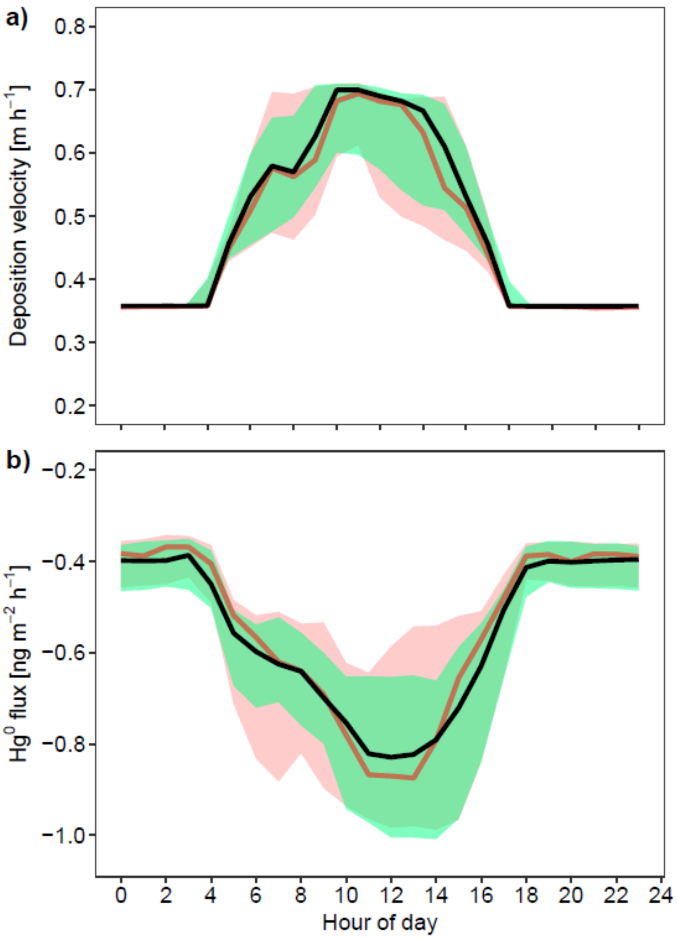

Figure S6. Diel pattern of the Hg^0 deposition velocity and Hg^0 flux measured with the micrometeorological approach. The Hg^0 deposition velocities (a) and the net Hg^0 flux (b) were calculated based on the arbitrarily selected value $R_{c,min} = \frac{1}{5} R_{c,max}$. Black lines with green areas show data obtained from the main instrument at 18.8 m height, red lines with red areas show data from 10.1 m height. Colored areas show the interquartile ranges ($Q_{0.25}$ to $Q_{0.75}$). The data were obtained from 15–28 April 2016.

References

(1) Agnan, Y.; Le Dantec, T.; Moore, C. W.; Edwards, G. C.; Obrist, D. New constraints on terrestrial surface–atmosphere fluxes of gaseous elemental mercury using a global database. *Environ. Sci. Technol.* **2016**, *50*, 507–524. https://doi.org/10.1021/acs.est.5b04013.

(2) Gustin, M. S.; Ericksen, J. A.; Schorran, D. E.; Johnson, D. W.; Lindberg, S. E.; Coleman, J. S. Application of Controlled Mesocosms for Understanding Mercury Air-Soil-Plant Exchange. *Environ. Sci. Technol.* **2004**, *38*, 6044–6050. https://doi.org/10.1021/es0487933.

(3) Stamenkovic, J.; Gustin, M. S. Evaluation of use of EcoCELL technology for quantifying total gaseous mercury fluxes over background substrates. *Atmos. Environ.* **2007**, *41*, 3702–3712. https://doi.org/10.1016/j.atmosenv.2006.12.037.

(4) Stamenkovic, J.; Gustin, M. S.; Arnone, J. A.; Johnson, D. W.; Larsen, J. D.; Verburg, P. S. J. Atmospheric mercury exchange with a tallgrass prairie ecosystem housed in mesocosms. *Sci. Tot. Environ.* **2008**, *406*, 227–238. https://doi.org/10.1016/j.scitotenv.2008.07.047.

(5) Eckley, C. S.; Gustin, M.; Lin, C.-J.; Li, X.; Miller, M. B. The influence of dynamic chamber design and operating parameters on calculated surface-to-air mercury fluxes. *Atmos. Environ.* **2010**, *44*, 194–203. https://doi.org/10.1016/j.atmosenv.2009.10.013.

(6) Eckley, C. S.; Tate, M. T.; Lin, C.-J.; Gustin, M.; Dent, S.; Eagles-Smith, C.; Lutz, M. A.; Wickland, K. P.; Wang, B.; Gray, J. E.; Edwards, G. C.; Krabbenhoft, D. P.; Smith, D. B. Surface-air mercury fluxes across western North America: A synthesis of spatial trends and controlling variables. *Sci. Total Environ.* **2016**, *568*, 651–665. https://doi.org/10.1016/j.scitotenv.2016.02.121.

(7) Gustin, M. S.; Lindberg, S.; Marsik, F.; Casimir, A.; Ebinghaus, R.; Edwards, G.; Hubble-Fitzgerald, C.; Kemp, R.; Kock, H.; Leonard, T.; London, J.; Majewski, M.; Montecinos, C.; Owens, J.; Pilote, M.; Poissant, L.; Rasmussen, P.; Schaedlich, F.; Schneeberger, D.; Schroeder, W.; Sommar, J.; Turner, R.; Vette, A.; Wallschlaeger, D.; Xiao, Z.; Zhang, H. Nevada STORMS Project: Measurement of mercury emissions from naturally enriched surfaces. *J. Geophys. Res.-Atmos.* **1999**, *104*, 21831–21844. https://doi.org/10.1029/1999JD900351.

(8) Zoo Zurich. *Zoojahr mit vielen Höhepunkten*. Zoo Zürich. 23. Januar **2017**, https://www.zoo.ch/de/medien/medienmitteilung/zoojahr-mit-vielen-hoehepunkten?language=de [last access: 12.08.2025]

(9) Guyton, A. C.; Hall, J. E. *Textbook of Medical Physiology*, 13th ed.; Elsevier, **2016**.
